# Impact of PET/CT among patients with suspected mycotic aortic aneurysms

**DOI:** 10.1371/journal.pone.0258702

**Published:** 2021-10-19

**Authors:** Lars Husmann, Martin W. Huellner, Hannes Gruenig, Nadia Eberhard, Carlos A. Mestres, Zoran Rancic, Barbara Hasse

**Affiliations:** 1 Department of Nuclear Medicine, University Hospital of Zurich, University of Zurich, Zurich, Switzerland; 2 Division of Infectious Diseases and Hospital Epidemiology, University Hospital of Zurich, University of Zurich, Zurich, Switzerland; 3 Clinic for Cardiac Surgery, University Hospital of Zurich, University of Zurich, Zurich, Switzerland; 4 Clinic for Vascular Surgery, University Hospital of Zurich, University of Zurich, Zurich, Switzerland; Ente Ospedaliero Cantonale, SWITZERLAND

## Abstract

**Purpose:**

To determine the impact of ^18^F-fluorodeoxyglucose (FDG) positron emission tomography/computed tomography (PET/CT) on clinical management in patients with suspected mycotic aortic aneurysms (MAA).

**Materials and methods:**

For this observational cohort study 101 PET/CT were acquired in 50 patients, thereof 50 for the initial diagnosis/baseline scan, 51 for follow-up. Impact on patient management was defined in three categories: PET/CT results were “confirmed” (by clinical follow-up), “suspected” (conclusive, not confirmed), or “misleading” (proven wrong by follow-up). For clinical follow-up patient data were recorded at the time of imaging, and at the latest recorded clinical visit. It included patient demographics, clinical information, laboratory data, results of microbiology and other diagnostic procedures, information about treatment, and patient’s general health condition.

**Results:**

In four patients (8%) no clinical follow-up was feasible, the other 46 patients were clinically followed for a median of 898 days (IQR 320–4105). The combined evaluation of all 101 PET/CT demonstrated an impact on patient management in 78,5% of cases (48,5% confirmed, 30% suspected). Results of 21,5% of the PET/CT examinations were misleading. Respective values at baseline and at follow-up were: impact on patient management in 82% and 74,5% (70% and 27.5% confirmed, and 12% and 47% suspected), misleading cases in 18% and 25.5%.

**Conclusion:**

In MAA, PET/CT has a high impact on patient management, which is more pronounced with baseline than with follow-up examinations. However, PET/CT results may be misleading in a smaller proportion of cases.

## Introduction

The impact of ^18^F-fluorodeoxyglucose (FDG) positron emission tomography/computed tomography (PET/CT) on clinical management of cancer patients based on a large prospective data registry has recently shown that physicians change their intended management in 37% of cases after PET/CT [1]. Comparable data for the impact of PET/CT in infectious diseases is scarce. For example, Leroy-Freschini et al. [2] described an impact of PET/CT in the management of immunocompromised patients with invasive fungal infections in 55% of their patients at initial staging and in 46% at follow-up. Moreover, the detection of peripheral emboli with PET/CT in patients with endocarditis or cardiac device infections induced therapeutic modifications in 24–44% of patients [3]. For mycotic aortic aneurysms (MAA), the actual impact of PET/CT on patient management has not been evaluated, yet.

MAA are infectious arterial aneurysms, caused by microbial inoculation of aortic endothelium during bacteraemia [4], and which account for 0.7–4.5% of all aortic aneurysms [5]. Surgical and medical treatment of mycotic aortic aneurysms (MAA) is demanding. Evidence indicates that endovascular aortic repair (EVAR) of MAA may be an equivalent treatment option to open surgical treatment [6]. However, EVAR of MAA inevitably leads to secondary vascular graft infections, requiring long-term antimicrobial therapy.

Regarding imaging of MAA, recent publications have demonstrated a high diagnostic accuracy of PET/CT in the detection of MAA [7], and the useful of PET/CT in therapy control of MAA [8]. Hence, the aim of the present study was to determine the impact of PET/CT on clinical management of patients with suspected MAA.

## Materials and methods

### Study design and definitions

Two different patient groups were included: i) prospective patients aged 18 years or older with suspected MAA and open and/or endovascular surgery enrolled in the Vascular Graft Cohort Study (VASGRA), and ii) retrospective patients with suspected MAA, who were examined with PET/CT between the years 2005 and 2018. The study was approved by the local ethics committee, namely the Kantonale Ethikkomission Zürich (protocol number 2018–01904), and we obtained written informed consent from all participants who were either prospectively enrolled or examined between the years 2016 and 2018; for subjects scanned between the years 2005 and 2015, written informed consent was waived due to retrospective inclusion by the local ethics committee, namely the Kantonale Ethikkomission Zürich (protocol number 2018–01904). All procedures were in accordance with the 1964 Helsinki declaration and its later amendments.

Patient demographics are described in Table 1 and S1 Table. Consecutively and prospectively enrolled patients had PET/CT scans at baseline, during follow-up on antimicrobial therapy, and at the end of antimicrobial treatment. In some patients, another control PET/CT three months after the end of antimicrobial therapy was acquired. For all retrospectively enrolled patients, PET/CT was acquired depending on the clinical condition of the patient. A chart review at each time of imaging, and at the last recorded clinical visit was performed. It included patient demographics, clinical information, laboratory and microbiological data, results from other diagnostic procedures, and information about treatment.

**Table 1:** Patient demographics at the time of the baseline PET/CT (total number of PET/CT examinations n = 101).

Number of patients, n (%)	50 (100)
Median age, years (IQR)	64 (56–85)
Male gender, n (%)	41 (82)
Diabetes mellitus, n (%)	9 (18)
Renal insufficiency ^1^, n (%)	23 (46)
Smoking/history of smoking, n (%)	25 (50)
Median C-reactive protein at time of imaging, mg/L (IQR)	69 (17–279)
Median WBC, G/L (IQR)	8.7 (6.7–19.5)
Number of confirmed mycotic aneurysms, n (%)	22 (44)

Abbreviations: PET/CT: positron emission tomography/computed tomography; CE: contrast-enhanced; IQR: interquartile range; WBC: white blood cell count.

Note:

^1^ Defined as glomerular filtration rate < 50 ml/min.

According to the MAGIC criteria [9] a secondary vascular graft infection was diagnosed if a stentgraft was placed in an infected area. In case of a native infected vessel, MAA was diagnosed in an overall appraisal of clinical presentation, laboratory and imaging [6, 10].

### Imaging data acquisition

Five different types of PET/CT scanners were used in our institution during the 15-year study period (i.e., Discovery ST16, Discovery VCT, Discovery MI, Discovery 690 and Discovery 710, all GE Healthcare, Waukesha, WI). All examinations were performed with the following imaging standards: a) PET/CT scan range at least from the vertex of the skull to the mid-thighs, b) non-enhanced CT scans for attenuation correction, c) all patients fasted for at least 4 hours and had no insulin injections within four hours before to the FDG administration, d) body-weight adapted intravenous injection of FDG after supine resting for a standardized uptake time of 60 minutes.

Additional contrast-enhanced CT of the chest and/or abdomen was performed as part of the PET/CT examination in a subset of patients, using a tube voltage of 120 kV and a tube current–time product of 100–320 mAs. CT was timed for imaging at arterial phase and portal venous phase after intravenous injection of 80 ml of iodinated contrast medium (Visipaque^®^ 320, GE Healthcare, Waukesha, WI).

All imaging data sets were analysed independently by two double board-certified radiologists and nuclear medicine physicians with 12 and 13 years of experience in hybrid imaging on a AW workstation version 4.7 (GE Healthcare Biosciences, Pittsburgh, PA). Primary and secondary diagnoses (e.g., infectious foci not in the vicinity of the aorta or other relevant findings such as malignancy) were documented. A consensus reading was performed if results differed.

### Impact on patient management

Impact on patient management was defined by three categories: “confirmed”, “suspected” and “misleading”. These were defined as follows:

Confirmed impact was noted if the PET/CT report conclusively answered the referral question or detected a relevant additional incidental finding, which was previously unknown to the referring physicians and changed patient management. All PET/CT findings, categorized as “confirmed”, had to be confirmed by clinical follow-up and/or other diagnostic modalities (e.g. PET-positive MAA confirmed by microbiology, or incidentally diagnosed cancer confirmed by histology).

Suspected impact was noted, if the PET/CT report either clearly answered the referral question or described an unsuspected and presumably relevant finding, however, the “suspected” impact could not be confirmed by other diagnostic modalities or by follow-up.

A misleading impact was noted, if the PET/CT report either clearly answered the referral question or described an incidental finding, but the findings were proven wrong either by follow-up or other diagnostic modalities.

Notably, if more than one referral question was to be answered by PET/CT, impact on management was noted if the main question was answered. In detail, if the referral question was just “infectious aneurysm?”, impact was noted, if PET/CT correctly confirmed or correctly ruled out the suspicion of MAA. However, if the question was “infectious aneurysm or other infectious foci?”, impact was only noted if PET/CT correctly described a particular site of infection; if PET/CT did not identify a particular site of infection, no impact on management was noted. The latter was the case, because the patient clinically had an infection, and hence the PET/CT scan must have been considered false negative. Furthermore, if the referral question was just “follow-up” of a known site of infection, impact on management was noted, if PET/CT results (e.g. decrease of FDG-activity) elicited a change in patient treatment (e.g., termination of antimicrobial treatment).

### Statistical analyses

Statistical analysis was performed using commercially available software (Stata, Version 16, StataCorp, College Station, Texas). Variables were expressed as median and IQR (25th, 75th percentiles) or percentages. One-sided Fisher`s exact tests were performed to determine differences in impact on patient management with regard to the referral question for the initial PET/CT. A *P*-value of < .05 was considered to indicate statistical significance, and a *P*-value between.05 and.1 was considered to indicate a statistical trend.

## Results

### Patient population

**Fig 1:**
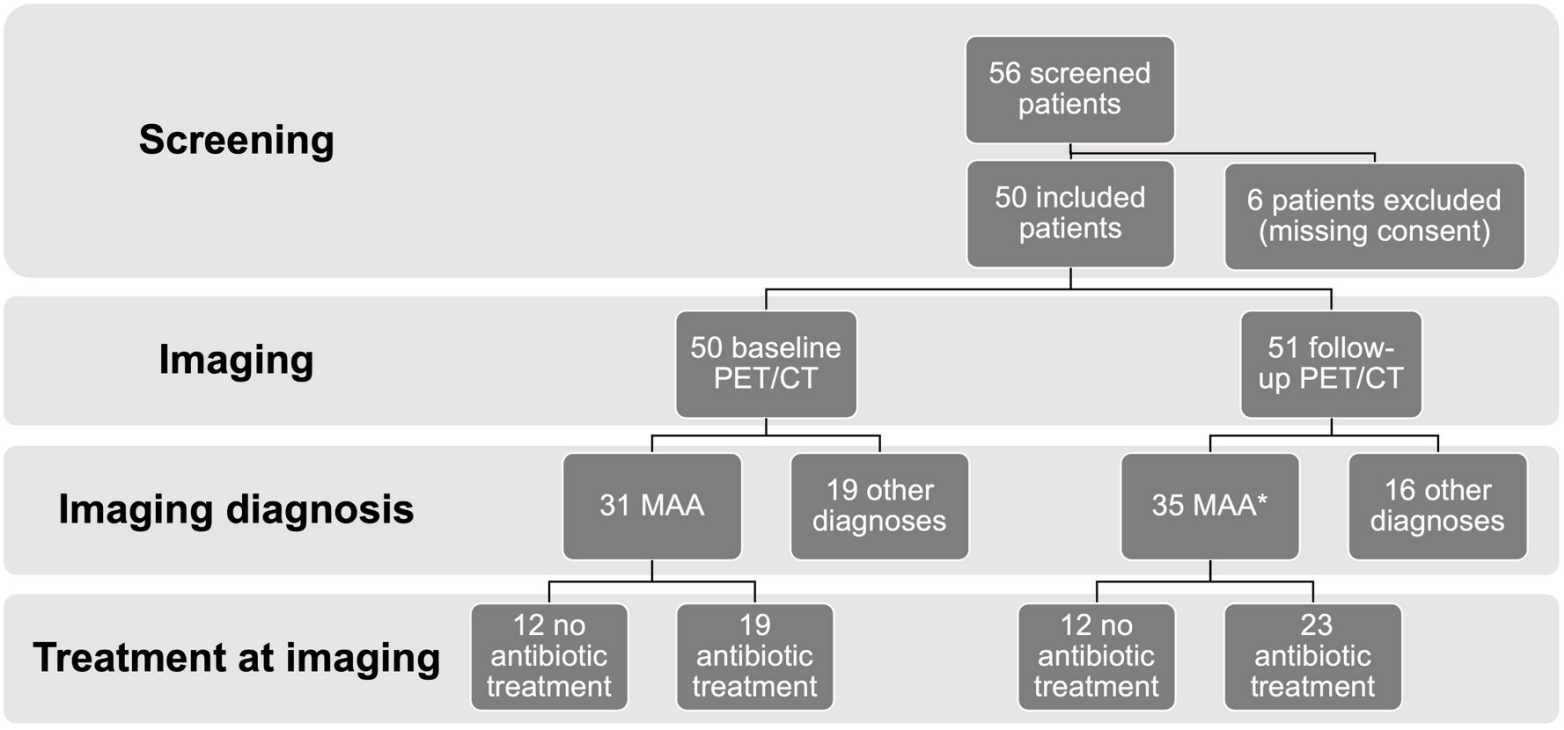
Flow diagram. Flow diagram displaying the numbers of patients screened, excluded, and included in the final study for suspected mycotic aortic aneurysms (MAA) in the first section. The second section shows the number of baseline and follow-up PET/CT (positron emission tomography/computed tomography) performed. Imaging diagnoses at baseline PET/CT are given in the third section, as well as the number of confirmed MAA at follow up (* notably, repetitive imaging in some patients with MAA accounts for the higher number as compared to baseline). Finally, the number of patients treated with antibiotics at the time of imaging is given in the bottom section.

Six patients were excluded because no written consent was signed (Fig 1). The final patient population consisted of 50 patients, whereof 15 patients (30%) were prospectively enrolled (part of the *Blinded for Review* cohort as described in the Methods sections), while 35 (70%) patients were retrospectively included in the study population. Twenty-two patients had a MAA, 15 were confirmed by blood culture, five by biopsy or in resected tissue; only in two cases blood culture and/or biopsy was negative, and MAA was diagnosed in an overall appraisal of clinical presentation, laboratory and imaging.

One hundred and one PET/CT scans were acquired, 50 for the initial diagnosis/baseline scan, and 51 follow up scans (22 for the first, 13 for the second, nine for the third, five for the fourth, and two for the fifth follow-up PET/CT examination). In 28 examinations (28%), an additional contrast-enhanced CT of the chest and/or the abdomen was acquired as part of the PET/CT scan (10 baseline scans, 10 first, three second, three third, and two forth PET/CT follow-up scans).

Completeness of follow-up was 92% as in four patients information could not be collected. Three patients were referred from other institutions and lost to follow-up, one patient decided to refrain from any further treatment. The remaining 46 patients (92%) were clinically followed for a median of 898 days (IQR 320–4105) after their baseline PET/CT and for a median of 429 days (IQR 82–4105) after their last PET/CT examination.

### Baseline PET/CT

Fifty baseline PET/CT examinations were performed. The clinical reasons for referral to the baseline PET/CT (n = 50) were (multiple reasons for referral possible): signs of MAA (n = 38), infectious foci in general (n = 16), vascular graft infection (n = 8), signs of aortitis (n = 4), lymphoma (n = 4), staging in bronchial carcinoma (n = 1), staging in gastric cancer (n = 1), restaging in urinary bladder cancer (n = 1), and question for Ormond’s disease (n = 1). The clinical symptoms were (multiple symptoms possible): abdominal pain (n = 21), chest pain (n = 6), leg pain (n = 2), back pain (n = 2), headache (n = 1), fever (n = 15), no symptoms (n = 10).

**Fig 2:**
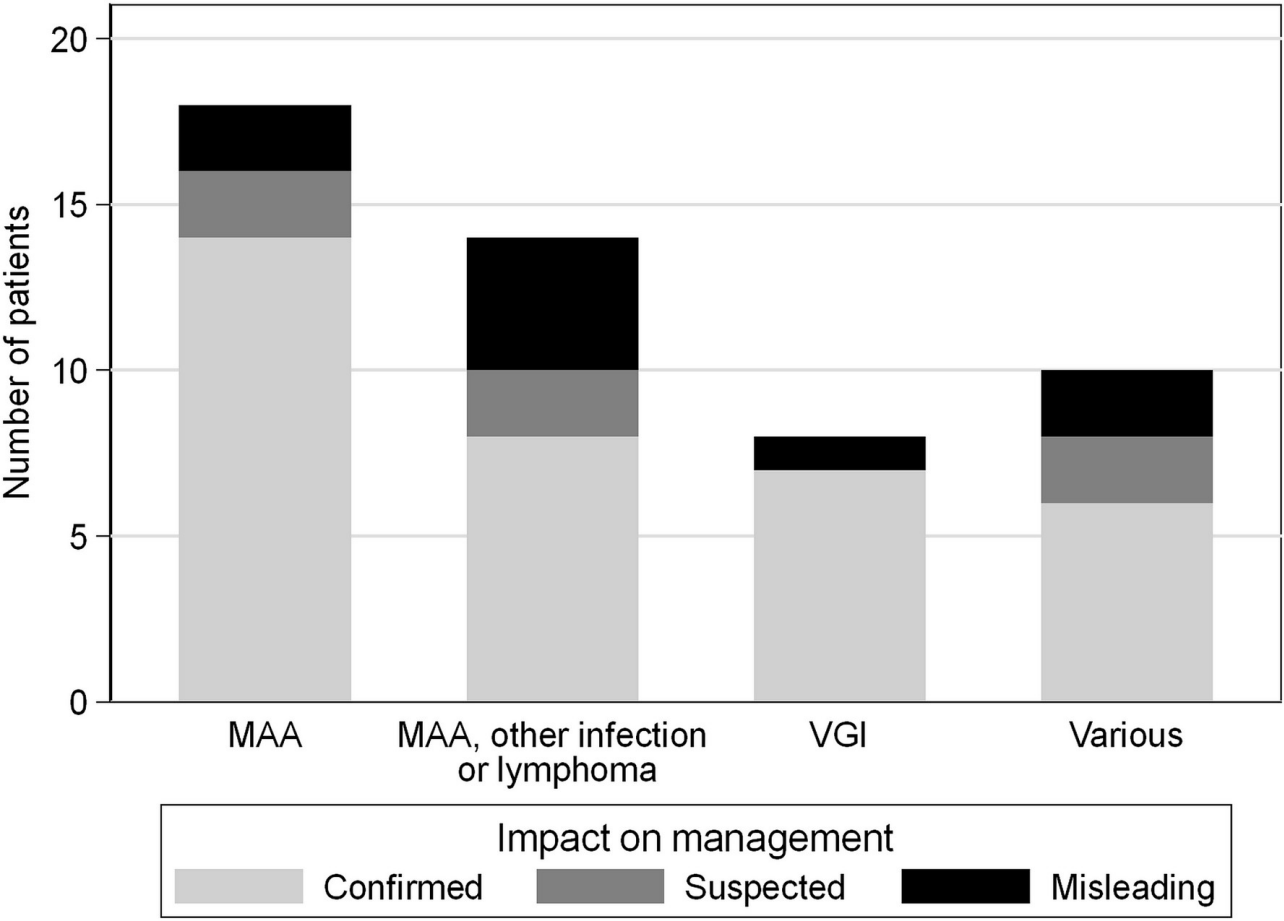
Number of baseline PET/CT with impact on patient management. Bar graph of all baseline PET/CT examinations in 50 patients demonstrating the number of examinations with impact on patient management (confirmed: light gray bars; suspected: dark gray bars) and the number of misleading examinations (black bars) with regard to different referral questions for PET/CT (first bar: mycotic aortic aneurysm (MAA); second bar: MAA, other infection or lymphoma; third bar: vascular graft infection (VGI); fourth bar: various referral questions).

**Fig 3:**
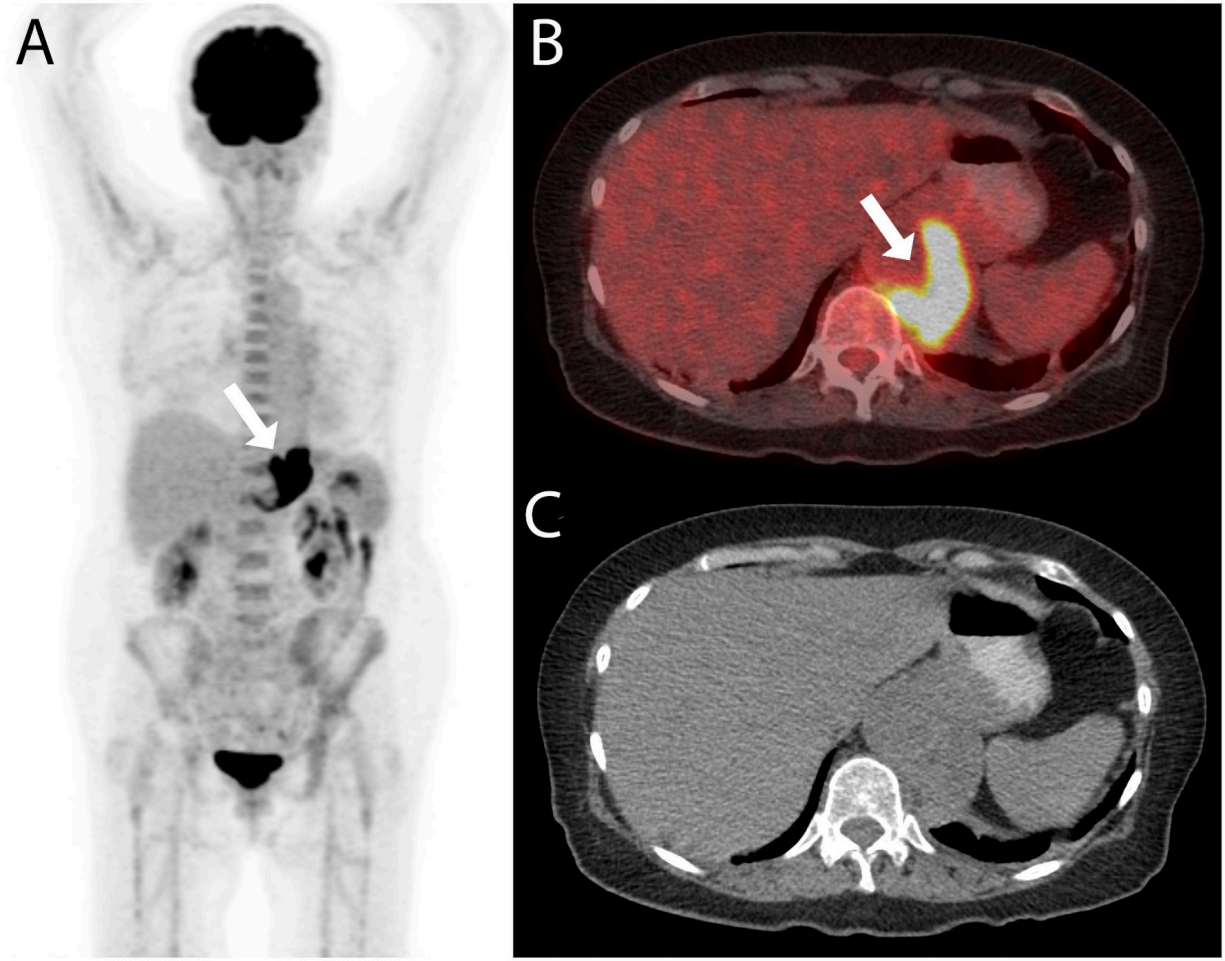
PET/CT with confirmed impact on management. A 71-year old woman was referred for PET/CT for staging of gastric cancer and with the question for an infectious aneurysm. The PET/CT examination (A: maximum intensity reconstruction, B: fused axial PET/CT image, and C: axial non-enhanced CT image) showed a FDG-avid mass adjacent to the abdominal aorta (white arrows in A and B). Both readers correctly rated the finding to be an infectious aneurysm; no gastric cancer was detected. The diagnosis was confirmed after biopsy (MAA caused by *Coxiella burnetii*) and the patient was treated with endovascular aortic repair and partial gastrectomy and lymphadenectomy. The patient was treated with antibiotics for 784 days and did not show any sign of recurrence at the last clinical visit 293 days after the end of antimicrobial therapy.

**Fig 4:**
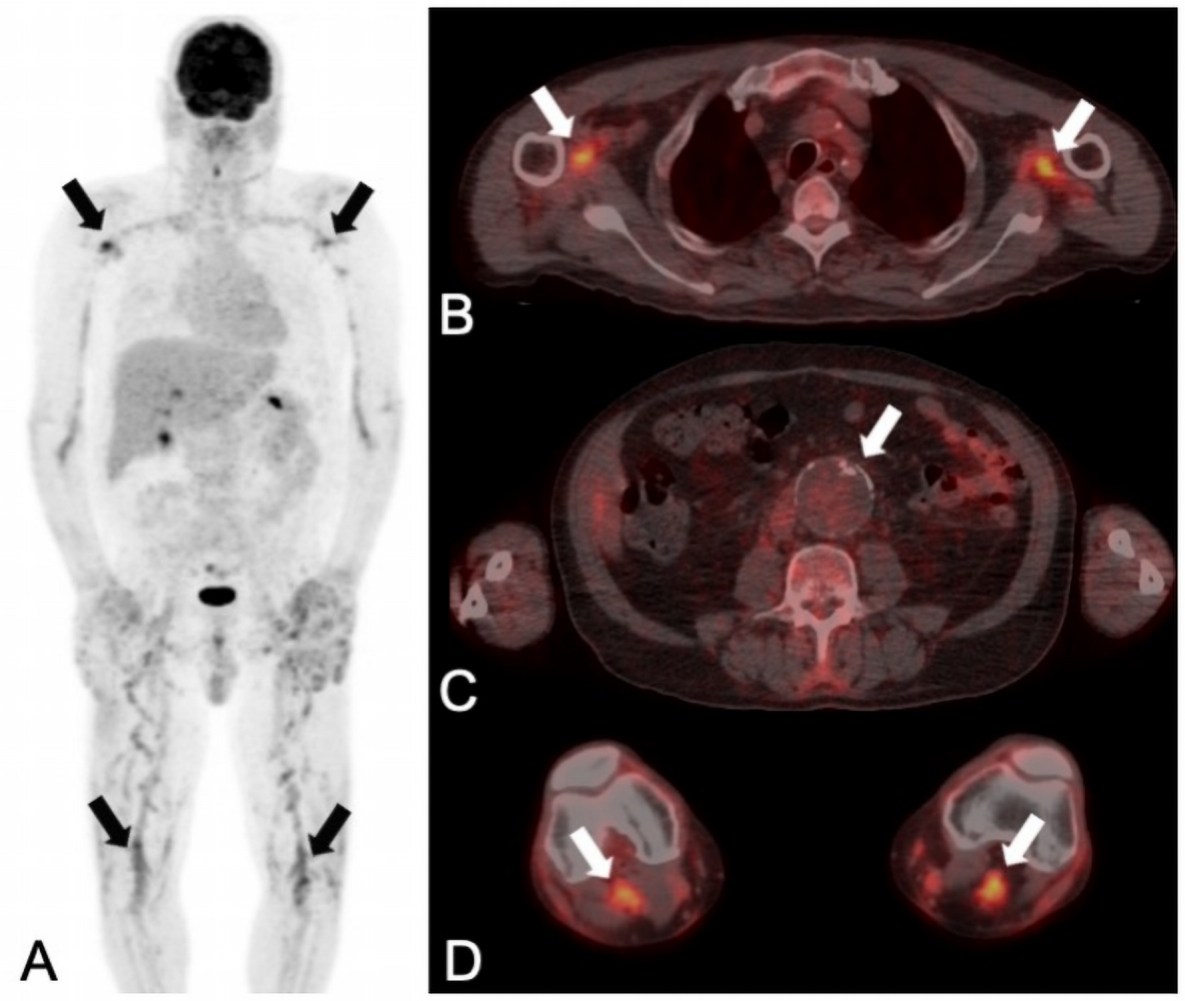
PET/CT with confirmed impact on management of an incidental finding. A 61-year old man was presented with recurrent episodes of fever and an elevated C-reactive protein level (154 mg/L). A PET/CT with the question for mycotic aortic aneurysm (MAA) or recurrence of lymphoma was acquired (A: maximum intensity reconstruction, B, C and D: fused axial PET/CT images). Lymphoma recurrence and MAA were correctly ruled out by both readers (no increased uptake in the abdominal aneurysm (white arrow in C)), as confirmed by long-term clinical follow-up. As an incidental finding with impact on patient management, PET/CT detected arteritis of the peripheral arteries (as demonstrated in the axillary (upper black arrows in A and white arrows in B) and popliteal arteries (lower black arrows in A and white arrows in D)). At the last clinical visit (826 days after PET/CT) the patient had continuous therapy with glucocorticoids. The patient was doing fine with no clinical signs of recurrent lymphoma, infection or inflammation.

At baseline, 32/50 (64%) PET/CT examinations answered the referral question conclusively (Fig 2), and all of these findings were confirmed by follow-up, i.e. 14 MAA (confirmed by blood culture (n = 10), or biopsy (n = 4); Fig 3), 10 rule-outs, five graft infections, one lymphoma, one periodontitis, and one arteritis (Fig 4) (Tables 2–5).

**Table 2:** Impact on patient management of baseline PET/CT scans with the referral question: Mycotic aortic aneurysm (n = 18).

Pat	Age	Main findings in PET/CT	Impact on management
15	48	MAA	Confirmed: treatment MAA
22	71	MAA	Misleading: treatment aneurysm“prerupture”
24	64	FDG-negative aneurysm	Confirmed: rule-out MAA
26	80	Pneumonia	Confirmed: treatment pneumonia
27	64	FDG-negative aneurysm	Suspected: rule-out MAA, VGI on follow-up
29	48	MAA	Misleading: no treatment of suspected activated plaques
30	81	MAA and bowel tumor	Confirmed: treatment MAA and colorectal adenoma
31	75	FDG-negative aneurysm	Suspected: no follow-up
32	76	MAA	Confirmed: treatment MAA
37	70	FDG-negative aneurysm	Confirmed: rule-out MAA
39	69	MAA	Confirmed: treatment MAA
41	84	FDG-negative aneurysm	Confirmed: rule-out MAA
43	52	MAA	Confirmed: treatment MAA
44	40	MAA	Confirmed: treatment MAA
51	61	MAA, spondylodiscitis	Confirmed: treatment MAA, spondylodiscitis
52	73	FDG-negative aneurysm	Confirmed: rule-out MAA
54	85	MAA and bowel tumor	Confirmed: treatment MAA
56	68	MAA	Confirmed: treatment MAA
			**14 confirmed, 2 suspected, 2 misleading**

Abbreviations: FDG: ^18^F-fluorodeoxyglucose; MAA: mycotic aortic aneurysm; Pat: Patient identification number; PET/CT: positron emission tomography/computed tomography; VGI: Vascular graft infection.

**Table 3:** Impact on patient management of baseline PET/CT scans with the referral question: MAA, other infection or lymphoma (n = 14).

Pat	Age	Main findings in PET/CT	Impact on management
02	62	MAA	Confirmed: treatment MAA
04	64	MAA	Confirmed: treatment MAA
12	64	MAA	Misleading: treatment inflammatory aneurysm
14	62	MAA	Misleading: treatment inflammatory aneurysm
16	58	MAA	Misleading: treatment inflammatory aneurysm
17	46	Lymphoma	Suspected: no follow-up
19	61	Arteritis	Confirmed: treatment arteritis
23	54	MAA	Misleading: no treatment of suspected inflammatory aneurysm
38	57	Periodontitis	Confirmed: treatment periodontitis
46	76	Endocarditis	Suspected: no follow-up
47	66	MAA	Confirmed: treatment MAA
48	61	Pneumonia	Confirmed: treatment pneumonia
53	54	Lymphoma	Confirmed: treatment lymphoma, enteritis
55	47	FDG-negative aneurysm	Confirmed: rule-out MAA
			**8 confirmed, 2 suspected, 4 misleading**

Abbreviations: FDG: ^18^F-fluorodeoxyglucose; MAA: mycotic aortic aneurysm; Pat: Patient identification number; PET/CT: positron emission tomography/computed tomography.

**Table 4:** Impact on patient management of baseline PET/CT scans with the referral question: Vascular graft infection (n = 8).

Pat	Age	Main findings in PET/CT	Impact on management
03	58	MAA/VGI	Confirmed: treatment VGI
06	74	Pneumonia, MAA/VGI	Confirmed: treatment VGI
07	82	MAA	Confirmed: treatment MAA
25	50	MAA/VGI	Confirmed: treatment VGI
33	56	MAA/VGI	Confirmed: treatment VGI
35	48	MAA/VGI	Misleading: no treatment of necrotic aneurysm
49	61	FDG-negative graft	Confirmed: rule-out VGI
50	56	Pneumonia	Confirmed: treatment pneumonia
			**7 confirmed, 1 misleading**

Abbreviations: FDG: ^18^F-fluorodeoxyglucose; MAA: mycotic aortic aneurysm; Pat: Patient identification number; PET/CT: positron emission tomography/computed tomography; VGI: Vascular graft infection.

**Table 5:** Impact on patient management of baseline PET/CT scans with the various referral questions (n = 10).

Pat	Age	Referral Question	Main findings PET/CT	Impact on management
05	70	MAA, aortitis?	MAA	Confirmed: treatment MAA
45	41	MAA, aortitis?	Pneumonia	Confirmed: treatment pneumonia
36	55	MAA, aortitis?	FDG-negative aneurysm	Confirmed: rule-out MAA, aortitis
18	65	MAA, lymphoma, Ormond`s disease?	Lymphoma	Suspected: no follow-up
34	71	Aortitis, other infection?	MAA	Confirmed: treatment MAA
01	69	VGI, other infection?	MAA/VGI	Confirmed: treatment VGI
21	65	Cancer restaging. MAA, infection?	MAA	Misleading: no treatment of suspected arteritis after radiation
40	71	Cancer restaging. MAA, infection?	MAA	Confirmed: treatment MAA
42	69	Cancer staging	No metastasis, MAA	Misleading: treatment of bronchial carcinoma, no treatment of abdominal aneurysm
28	73	Cancer restaging	Lymphoma and MAA	Suspected: no follow-up
				**6 confirmed, 2 suspected, 2 misleading**

Abbreviations: FDG: ^18^F-fluorodeoxyglucose; MAA: mycotic aortic aneurysm; Pat: Patient identification number; PET/CT: positron emission tomography/computed tomography; VGI: Vascular graft infection.

Nine (18%) of the baseline PET/CT could clearly answer the referral question, however, findings could not be confirmed, due to missing or incomplete follow-up (in these cases the impact of PET/CT on patient management was defined as “suspected”), i.e. one MAA, three rule-outs of MAA, one vascular graft infection (VGI), three lymphomas, and one endocarditis (Tables 2–5).

In nine (18%) cases, PET/CT findings were “misleading” since they were proven wrong by follow-up, i.e. in all of these cases, an MAA was suspected in PET/CT but not confirmed. The final diagnosis in these cases were: five inflammatory aneurysms, one necrotic aneurysm, one aneurysm “prerupture” (non-ruptured abdominal aortic aneurysm in a hemodynamically unstable patient), one aneurysm with a metabolically active plaque, and one aneurysm, in which the increased metabolic activity could not be explained, but long-term clinical follow-up ruled out infection (Tables 2–5).

Incidental findings were detected in 12 patients, of these 11 were confirmed (five pneumonias, one lymphoma, two colorectal adenomas, one spondylodiscitis, one arteritis, one periodontitis), while one was only suspected (i.e. suspected lymphoma, lost to follow-up). In three patients, the incidental finding was the reason why PET/CT had an impact on patient management (i.e., two confirmed pneumonias and one confirmed colorectal adenoma in three patients, one with an FDG-negative aneurysm and the other two with a suspected graft infection, both with insufficient follow-up for the latter). In all other cases (n = 10), PET/CT also had an impact on management since it correctly answered the referral question (e.g. in a patient with a confirmed MAA, the additionally detected spondylodiscitis was not rated as an additional impact, but just an overall impact of PET/CT on patient management). One examination had a confirmed impact on patient management but was also misleading (i.e. staging of bronchial carcinoma was correct, however, the incidentally detected and suspected MAA was not confirmed by clinical follow-up); thus the examination was deemed “misleading” (Tables 2–5).

Hence, baseline PET/CT had impact on patient management in 82% of cases (70% confirmed, and 12% suspected). PET/CT results were misleading in 18% of cases. With regard to the referral question, a trend (*P* = .077) towards higher frequencies of PET/CT examinations with confirmed impact on patient management (versus suspected and misleading results) was detected when the referral question was precise (i.e. MAA or VGI, Fig 2 and Tables 2 and 4) as opposed to other vaguer referral questions (Fig 2 and Tables 3 and 5).

### Follow-up PET/CT

All follow-up PET/CT examinations (n = 51) had diagnostic image quality. Follow-up PET/CT scans were acquired for (multiple reasons for referral possible): therapy control of the MAA and/or graft infection (n = 37), spondylodiscitis (n = 3), psoas muscle abscess (n = 3), and arteritis (n = 1), question for vascular graft infection (n = 5) or other infectious foci (n = 5), restaging of lymphoma (n = 2) and urinary bladder cancer (n = 1), search for malignancy (n = 1), and reevaluation of an unclear colorectal lesion (n = 1).

**Fig 5:**
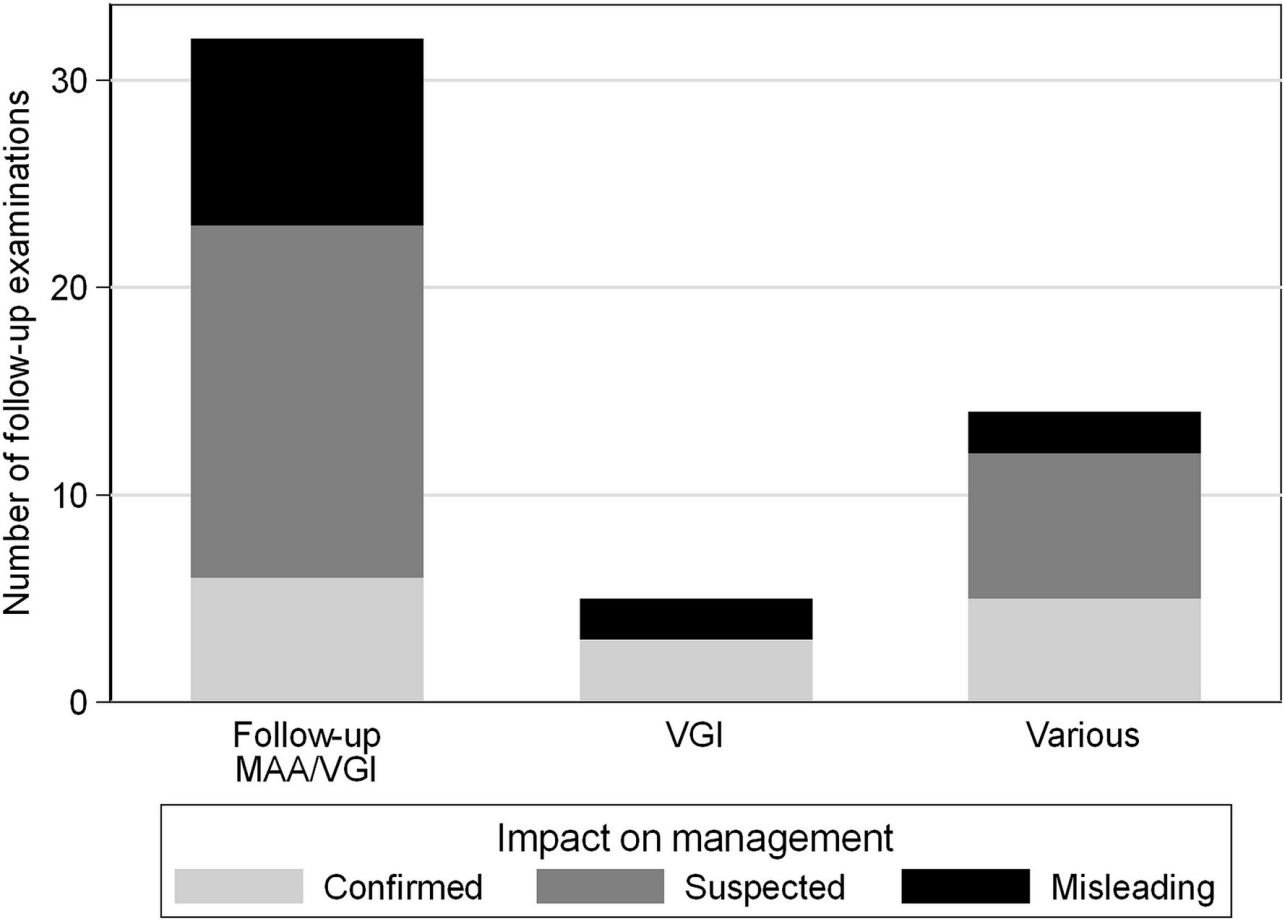
Number of follow-up PET/CT with impact on patient management. Bar graph of all follow-up PET/CT examinations (n = 51) demonstrating the number of examinations with impact on patient management (confirmed: light gray bars; suspected: dark gray bars) and the number of misleading examinations (black bars) with regard to different referral questions for PET/CT (first bar: follow-up of mycotic aortic aneurysm (MAA) or vascular graft infection (VGI); second bar: VGI; third bar: various referral questions).

At follow-up, 13 (25.5%) PET/CT examinations answered the referral question conclusively (Fig 5), and all findings were confirmed by follow-up, i.e. two MAA (both progressive and confirmed by blood culture), two VGI (one progressive and one newly diagnosed, one confirmed by biopsy, one by blood culture), one stable disease (PET/CT results lead to restart of antimicrobial treatment), and eight rule-outs.

Another 25 (49%) of the follow-up PET/CT scans could clearly answer the referral question, however findings could not be confirmed by other means or due to missing or incomplete follow-up (in these cases the impact of PET/CT on patient management was deemed “suspected”), i.e. stable VGI (n = 7), partial regression in metabolic activity of a VGI (n = 17), complete regression but missing follow-up (n = 1).

**Fig 6:**
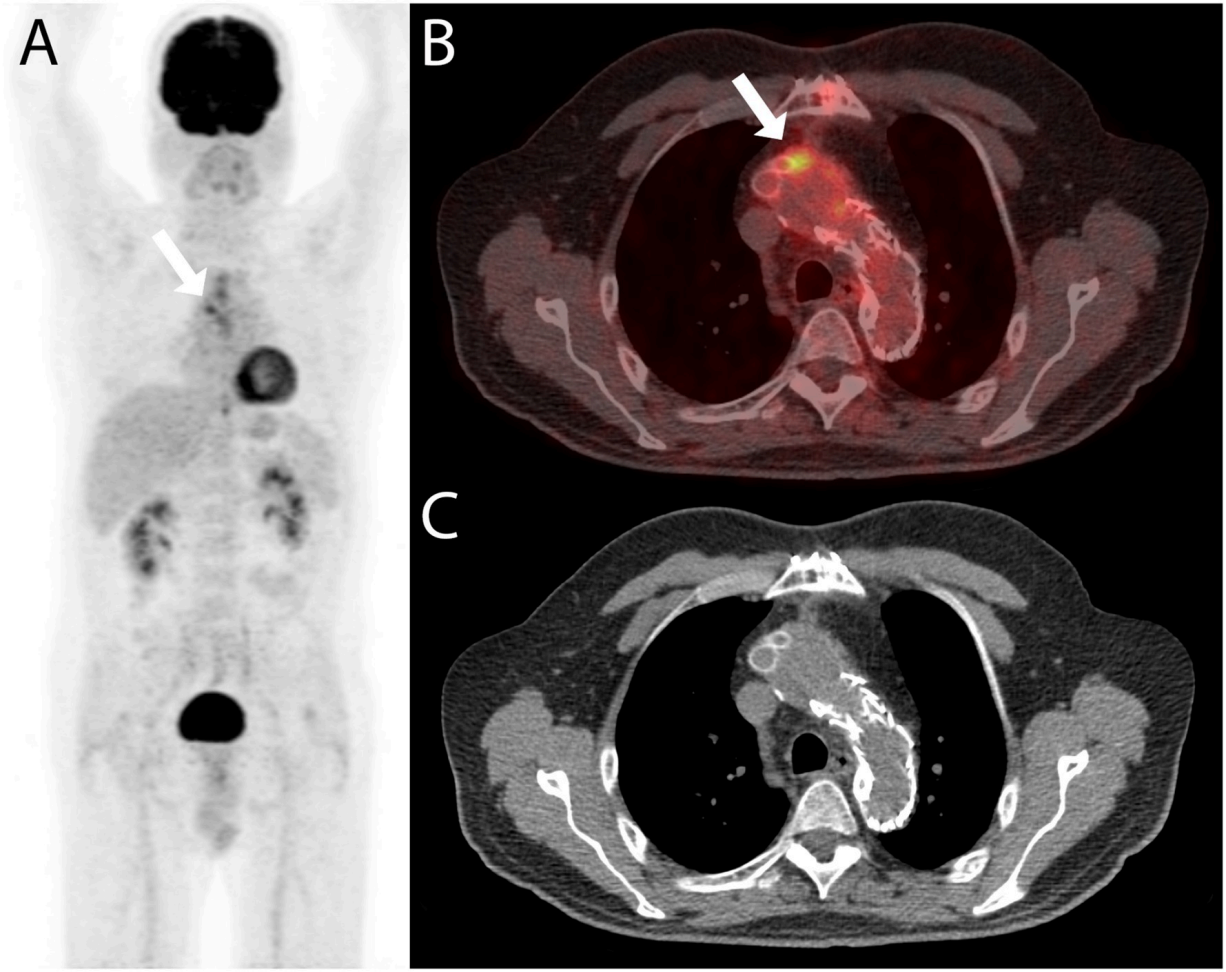
PET/CT with misleading diagnosis. The first follow-up PET/CT examination (A: maximum intensity reconstruction, B: fused axial PET/CT image, and C: axial non-enhanced CT image) of a 64-year old man was performed four months after the initial diagnosis of a mycotic aortic aneurysm, four months after the beginning of antimicrobial treatment and three months after thoracic endovascular aortic repair and supra-aortic debranching. Focal FDG accumulation adjacent to the graft (white arrows in A and B) was detected and rated positive for a remaining vascular graft infection by both readers. However, antimicrobial treatment was stopped 17 days after the PET/CT examination and no signs of recurrent infection was detected on clinical follow-up visits (the last one 667 days after the end of treatment). Therefore, PET/CT was rated “misleading”.

In 13 (25.5%) cases, PET/CT findings were “misleading” as they were proven wrong by follow-up. One PET/CT showed no pathologic FDG-uptake, but the patient had endocarditis. In the remaining 12 cases a VGI was suspected in PET/CT, but not confirmed (the FDG-positive findings in these cases were attributed to inflammatory aneurysms (n = 2) or remained unclear (n = 10) (possibly postoperative or granulomatous inflammatory reaction) (Fig 6).

Five incidental findings were detected in four examinations of four patients, all of them were confirmed (one pneumonia, two colorectal adenoma, one infected intestinal fistula, and one infected pancreatic cyst). Only in one follow-up examination the incidental finding was the reason why PET/CT had an impact on patient management, i.e. one patient with a stable VGI (rated as suspected impact) with newly detected fistulas and a new colorectal adenoma (finally rated as confirmed impact) (Tables 6–8).

**Table 6:** Impact on patient management of follow-up PET/CT scans with the referral question: Follow-up MAA/VGI (n = 32).

Pat	Age	Main findings in PET/CT	Impact on management
01	70	Increasing FDG-activity in MAA/VGI	Misleading: no change in treatment
02	62	Faintly/diffuse FDG-positive MAA/VGI	Confirmed: end of treatment
02	62	colon lesion	Confirmed: coloscopy
02	62	faintly FDG-positive MAA/VGI	Misleading: no treatment
02	64	faintly FDG-positive MAA/VGI	Misleading: no treatment
03	58	Decreasing FDG-activity in MAA/VGI	Suspected: no change in treatment
03	59	Decreasing FDG-activity in MAA/VGI	Suspected: no change in treatment
03	59	faintly FDG-positive MAA/VGI	Misleading: end of treatment
03	59	faintly FDG-positive MAA/VGI	Misleading: no treatment
05	70	Decreasing FDG-activity in MAA/VGI	Suspected: no change in treatment
05	71	Decreasing FDG-activity in MAA/VGI	Suspected: no change in treatment
07	82	Increasing FDG-activity in MAA	Confirmed: treatment MAA
07	82	Decreasing FDG-activity in MAA/VGI and spondylodiscitis	Suspected: no change in treatment
07	83	faintly FDG-positive MAA/VGI	Misleading: no treatment
15	49	Decreasing FDG-activity in MAA/VGI	Suspected: no change in treatment
25	51	Increasing FDG-activity in MAA/VGI	Confirmed: start of treatment
25	51	unchanged FDG-positive MAA/VGI	Suspected: no change in treatment
25	52	unchanged FDG-positive MAA/VGI	Suspected: no change in treatment
25	52	unchanged FDG-positive MAA/VGI	Suspected: no change in treatment
25	53	unchanged FDG-positive MAA/VGI	Suspected: no change in treatment
27	66	unchanged FDG-positive MAA/VGI	Suspected: no change in treatment
27	66	unchanged FDG-positive MAA/VGI	Suspected: no change in treatment
32	76	Decreasing FDG-activity in MAA/VGI	Suspected: no change in treatment
33	56	Decreasing FDG-activity in MAA/VGI	Suspected: no change in treatment
33	56	Decreasing FDG-activity in MAA/VGI	Suspected: no change in treatment
33	56	unchanged FDG-positive MAA/VGI	Misleading: end of treatment
33	57	Increasing FDG-activity in MAA/VGI	Misleading: no treatment
35	48	FDG-positive MAA/VGI	Misleading: end of treatment
35	48	FDG-negative MAA/VGI	Suspected: no follow-up
40	73	Decreasing FDG-activity in MAA/VGI	Suspected: no change in treatment
43	52	Increasing MAA	Confirmed: treatment MAA
44	41	Pneumonia, FDG-negative MAA/VGI	Confirmed: treatment pneumonia
			**6 confirmed, 17 suspected, 9 misleading**

Abbreviations: FDG: ^18^F-fluorodeoxyglucose; MAA: mycotic aortic aneurysm; Pat: Patient identification number; PET/CT: positron emission tomography/computed tomography; VGI: Vascular graft infection.

**Table 7:** Impact on patient management of follow-up PET/CT scans with the referral question: VGI (n = 5).

Pat	Age	Main findings in PET/CT	Impact on management
14	62	faintly FDG-positive MAA/VGI	Misleading: no VGI
16	59	VGI	Misleading: treatment of IAAA
27	65	VGI	Confirmed: treatment VGI
49	62	FDG-negative MAA/VGI	Confirmed: no treatment
54	85	FDG-negative MAA/VGI	Confirmed: no treatment
			**3 confirmed, 2 misleading**

Abbreviations: FDG: ^18^F-fluorodeoxyglucose; MAA: mycotic aortic aneurysm; Pat: Patient identification number; PET/CT: positron emission tomography/computed tomography; VGI: Vascular graft infection.

**Table 8:** Impact on patient management of follow-up PET/CT scans with the various referral questions (n = 14).

Pat	Age	Referral Question	Main findings PET/CT	Impact on management
44	40	Follow-up MAA/VGI, other infection?	Decreasing FDG-activity in MAA/VGI	Suspected: no change in treatment
44	47	Follow-up MAA/VGI, other infection?	No FDG-positive lesions	Misleading: death due to endocarditis 180 days after the PET/CT
04	64	VGI, other infection?	Faintly FDG-positive MAA/VGI	Misleading: end of treatment
16	59	Follow-up IAAA	Decreasing FDG-activity in IAAA	Suspected: no change in treatment
16	61	Follow-up IAAA	Decreasing FDG-activity in IAAA	Suspected: no change in treatment
51	62	Follow-up infections	Decreasing infectious foci	Suspected: no change in treatment
51	63	Follow-up infections	Decreasing infectious foci	Suspected: no change in treatment
51	64	Follow-up infections	Decreasing infectious foci	Suspected: no change in treatment
51	64	Follow-up infections	Decreasing infectious foci	Suspected: no change in treatment
51	66	Follow-up infections	Decreasing infectious foci	Confirmed: no recurrence
19	61	Cancer restaging	Colon lesion	Confirmed: coloscopy
19	62	Cancer restaging	No FDG-positive lesions	Confirmed: no recurrence of cancer or arteritis
21	65	Cancer restaging	No FDG-positive lesions	Confirmed: no recurrence of cancer
43	53	Cancer restaging	Pancreatic lesion	Confirmed: treatment infected pancreatic cyst
				**5 confirmed, 7 suspected, 1 misleading**

Abbreviations: FDG: ^18^F-fluorodeoxyglucose; IAAA: inflammatory abdominal arterial aneurysm; MAA: mycotic aortic aneurysm; Pat: Patient identification number; PET/CT: positron emission tomography/computed tomography; VGI: Vascular graft infection.

Hence, follow-up PET/CT had an impact on patient management in 74,5% of cases (27.5% confirmed, and 47% suspected). Follow-up PET/CT results were misleading in 25.5% (Fig 5, Tables 6–8).

### Baseline and follow-up PET/CT combined

The combined evaluation of all 101 PET/CT (50 baseline PET/CT, and 51 follow-up PET/CT) demonstrated an impact on patient management in 78,5% of cases (48,5% confirmed, and 30% suspected). Results of 21,5% of the PET/CT examinations were misleading.

## Discussion

The study assessed the impact of PET/CT on clinical management of patients with suspected MAA. In 101 PET/CT examinations of 50 patients, a high impact on patient management was demonstrated, which was more pronounced with baseline than with follow-up examinations. In a small subset of patients in the present study, PET/CT results were misleading.

PET/CT is considered as the imaging gold standard in the diagnosis and in therapy control of many tumors. Numerous studies have demonstrated the impact of PET/CT on patient management by either questioning the referring physicians prior and after PET/CT on their intended treatment approach [1], or by comparing the additional information of PET/CT to findings of conventional imaging [11]. If, for example, a single metastasis detected only by PET/CT but not by conventional imaging, leads to an upstaging of a patient, it may cause a possible shift of strategy from local to systemic treatment. The latter is an excellent definition for “impact of PET/CT on patient management” [11]. Furthermore, if PET/CT results in oncologic imaging are equivocal or doubted, they often may be confirmed by biopsy—an option which is not viable in many infectious diseases.

To the best of the knowledge, the present study is the first study to investigate the impact of PET/CT on patient management in MAA, and only a few other studies have evaluated the impact of PET/CT in other infections [3, 12–19]. A previous study analyzed the impact of PET/CT in the management of immunocompromised patients with invasive fungal infections [2], and defined impact by an influence in the diagnostic work-up at primary staging by assessing the extent of infection and targeting the diagnostic procedure, or by an influence on anti-fungal drug dosage or treatment withdrawal. In MAA, a comparable definition is difficult, since even the gold standard for the diagnosis of the disease is a combination of the overall appraisal of clinical presentation, laboratory results, and imaging [6]. Even more so, no consensus exists for determinants, or cut-offs values, which may justify the end of antimicrobial treatment in MAA. At present, treatment decisions in patients with MAA will always be based on a combination of results from different diagnostic tests and tools. Therefore, the impact of a single method (e.g., PET/CT) on patient management is almost impossible to differentiate from others. Hence, a new definition of “impact on management” for PET/CT in MAA was established. Impact was noted, when either the referral question to PET/CT was clearly answered, and clinical follow-up confirmed PET/CT results, or if and incidental finding (e.g., detection of a previously unknown site of infection or tumor) clearly changed the patient management.

By these means, the present study yields considerably higher impact of PET/CT on patient management in patients with suspected MAA as compared to previous studies with oncologic patients. A recent large prospective study [1] demonstrated a change of intended management in 37% of cancer patients after PET/CT, while the present study describes a confirmed impact of at least 70% at baseline PET/CT.

### Limitations

The newly introduced definition of “impact on management” challenges the comparability of the present study results to previous. However, as described above, previously used definitions for other diseases may not be easily transferred to the treatment of MAA, and no comparable previous data on the impact on management of PET/CT in MAA exist in the literature.

Furthermore, no PET/CT imaging criteria for diagnosing MAA were defined, as our study was retrospective, and at the time of image reading, no criteria existed in the literature. In our previous work [7], we could show, that a cut-off criterion “higher FDG-uptake in the aneurysm than in liver background” lead to a sensitivity of 100% in diagnosing MAA, but the criterion was hampered by a low specificity due to false-positive findings (e. g. in inflammatory aneurysms).

Finally, the present study population was heterogeneous, with varying numbers of follow-up PET/CT examinations, using different PET/CT scanner generations, as well as prospectively and retrospectively included patients. However, it represents a large study population with MAA examined with PET/CT. Nonetheless, further studies are required to confirm the results.

## Conclusion

In MAA, PET/CT has a high impact on patient management, which is more pronounced with baseline than with follow-up examinations. However, PET/CT results may be misleading in a subset of patients.

## Supporting information

S1 Table Raw data of all included patients. Abbreviations: Pat ID: Patient identification number; DM: Diabetes mellitus; Smoking: History of smoking; Renal insuff.: Renal insufficiency; AB: Antibiotic treatment; CRP: C-reactive protein; WBC: white blood cell count; PET/CT: positron emission tomography/computed tomography; na: not applicable; 1: yes; 0: no.
(DOCX)
