# Supplementary material for: Impact of PET/CT among patients with suspected mycotic aortic aneurysms
Source: PLoS One. 2021 Oct 19;16(10):e0258702. doi: 10.1371/journal.pone.0258702 (PMC8525767; doi:10.1371/journal.pone.0258702)
Supplement: S1 Table — Abbreviations: Pat ID: Patient identification number; DM: Diabetes mellitus; Smoking: History of smoking; Renal insuff.: Renal insufficiency; AB: Antibiotic treatment; CRP: C-reactive protein; WBC: white blood cell count; PET/CT: positron emission tomography/computed tomography; na: not applicable; 1: yes; 0: no. (DOCX) [file pone.0258702.s001.docx]

| **Pat ID** | **Age** | **Male** | **DM** | **Smoking** | **Renal insuff.** | **AB** | **CRP** | **WBC** | **Date PET/CT** | **Date last control** |
| --- | --- | --- | --- | --- | --- | --- | --- | --- | --- | --- |
| MYC001 | 69 | 1 | 0 | 1 | 1 | 1 | 4,6 | 7,8 | 02.09.16 | 16.04.18 |
| MYC002 | 62 | 1 | 0 | 1 | 0 | 1 | 199 | 19,5 | 24.07.12 | 17.09.14 |
| MYC003 | 58 | 1 | 0 | 1 | 0 | 1 | 7,3 | 13,9 | 26.01.17 | 05.02.19 |
| MYC004 | 64 | 1 | 0 | 0 | 0 | 1 | 27 | 8,97 | 20.03.15 | 14.06.17 |
| MYC005 | 70 | 1 | 1 | 0 | 1 | 0 | 122 | 9,1 | 17.09.13 | 12.07.14 |
| MYC006 | 74 | 1 | 1 | 0 | 1 | 1 | 85 | 6,95 | 22.04.16 | 10.05.16 |
| MYC007 | 82 | 1 | 0 | 1 | 0 | 1 | 95 | 10,2 | 21.09.17 | 16.01.19 |
| MYC012 | 64 | 1 | 0 | 0 | 0 | 0 | 47 | 3,8 | 05.05.17 | 28.11.18 |
| MYC014 | 62 | 1 | 0 | 1 | 1 | 0 | 9,2 | 5,89 | 01.11.17 | 23.11.18 |
| MYC015 | 48 | 1 | 0 | 1 | 1 | 1 | 145 | 10,7 | 10.11.16 | 27.06.18 |
| MYC016 | 58 | 1 | 1 | 1 | 1 | 1 | 44 | 7,39 | 27.02.15 | 15.12.17 |
| MYC017 | 46 | 0 | 0 | 0 | 1 | 1 | 79 | 14,5 | 13.07.18 | 27.08.18 |
| MYC018 | 65 | 1 | na | na | na | na | na | na | 11.01.17 | na |
| MYC019 | 61 | 1 | 1 | 1 | 1 | 1 | 154 | 15,9 | 09.06.16 | 13.09.18 |
| MYC021 | 65 | 1 | 0 | 1 | 0 | 1 | 212 | 5,6 | 17.02.15 | 17.10.18 |
| MYC022 | 71 | 1 | 0 | 1 | 0 | 0 | 11 | 9,3 | 24.06.15 | 08.08.18 |
| MYC023 | 54 | 1 | 0 | 0 | 0 | 0 | 23 | 6,3 | 23.02.15 | 20.10.17 |
| MYC024 | 64 | 0 | 0 | 1 | 0 | 0 | na | 6,3 | 25.02.14 | 10.10.18 |
| MYC025 | 50 | 1 | 0 | 0 | 1 | 0 | 17 | 8,72 | 23.06.14 | 06.12.18 |
| MYC026 | 80 | 1 | 0 | 1 | 0 | 1 | 137 | 8,8 | 12.08.14 | 01.11.14 |
| MYC027 | 64 | 1 | 1 | 1 | 1 | 0 | 14 | 7,6 | 19.08.14 | 27.01.19 |
| MYC028 | 73 | 1 | 1 | 0 | 0 | 0 | na | na | 23.09.14 | na |
| MYC029 | 48 | 1 | 0 | 1 | 0 | 0 | 12 | 10,3 | 20.01.14 | 18.05.18 |
| MYC030 | 81 | 0 | 0 | 1 | 1 | 1 | 140 | 10,7 | 05.03.14 | 14.04.14 |
| MYC031 | 75 | 0 | 0 | 0 | 1 | 0 | 16 | 8,2 | 22.10.14 | na |
| MYC032 | 76 | 1 | 0 | 0 | 0 | 1 | 69 | 7,5 | 07.01.13 | 29.07.16 |
| MYC033 | 56 | 1 | 0 | 1 | 0 | 1 | 11 | 13,2 | 28.02.12 | 27.06.18 |
| MYC034 | 71 | 1 | 1 | 0 | 0 | 0 | na | na | 26.04.13 | 26.06.13 |
| MYC035 | 48 | 1 | 0 | 0 | 0 | 1 | 27 | 5,58 | 23.07.13 | 18.12.13 |
| MYC036 | 55 | 1 | 0 | 0 | 0 | 1 | 121 | 18,8 | 21.02.13 | 28.02.17 |
| MYC037 | 70 | 0 | 0 | 1 | 0 | 0 | 96 | 8,71 | 04.10.12 | 18.05.14 |
| MYC038 | 57 | 1 | 0 | 0 | 1 | 1 | 10 | 6,63 | 06.01.11 | 18.05.18 |
| MYC039 | 69 | 0 | 0 | 0 | 0 | 1 | 34 | 11,96 | 02.03.11 | 20.03.12 |
| MYC040 | 71 | 0 | 0 | 0 | 1 | 1 | 31 | 9,53 | 21.06.11 | 02.06.14 |
| MYC041 | 84 | 0 | 0 | 0 | 1 | 1 | 93 | 6,68 | 26.04.11 | 19.07.11 |
| MYC042 | 69 | 1 | 0 | 0 | 0 | 0 | na | na | 28.06.11 | 01.12.11 |
| MYC043 | 52 | 1 | 1 | 0 | 0 | 0 | 100 | 10,5 | 24.06.10 | 22.03.11 |
| MYC044 | 40 | 1 | 0 | 0 | 0 | 1 | 146 | 6,8 | 13.12.10 | 04.09.18 |
| MYC045 | 41 | 1 | 0 | 1 | 0 | 1 | 19 | 9,78 | 14.01.09 | 19.04.17 |
| MYC046 | 76 | 1 | 0 | 1 | 1 | na | na | na | 02.04.09 | na |
| MYC047 | 66 | 1 | 1 | 0 | 1 | 1 | 93 | 10,7 | 16.01.09 | 28.01.09 |
| MYC048 | 61 | 1 | 0 | 1 | 1 | 1 | 279 | 7,34 | 28.04.09 | 18.05.09 |
| MYC049 | 61 | 1 | 0 | 0 | 0 | 1 | 23 | 5,34 | 02.02.09 | 25.10.17 |
| MYC050 | 56 | 1 | 0 | 1 | 1 | 0 | 273 | 10,28 | 06.08.09 | 19.01.18 |
| MYC051 | 61 | 1 | 0 | 1 | 1 | 0 | 10 | 6,71 | 18.11.08 | 26.03.14 |
| MYC052 | 73 | 1 | 0 | 1 | 0 | 0 | 13 | 5,41 | 28.12.07 | 23.10.13 |
| MYC053 | 54 | 0 | 0 | 1 | 1 | 1 | na | na | 07.05.07 | 02.08.18 |
| MYC054 | 85 | 1 | 0 | 0 | 1 | 1 | 126 | 5,18 | 30.08.07 | 17.01.13 |
| MYC055 | 47 | 1 | 0 | 0 | 0 | 0 | 253 | 10,4 | 06.10.06 | 17.12.09 |
| MYC056 | 68 | 1 | 0 | 1 | 1 | 1 | 234 | 8,2 | 13.10.05 | 08.10.10 |
